# Vitamin D3 constrains estrogen’s effects and influences mammary epithelial organization in 3D cultures

**DOI:** 10.1038/s41598-019-43308-1

**Published:** 2019-05-15

**Authors:** Nafis Hasan, Carlos Sonnenschein, Ana M. Soto

**Affiliations:** 1 Cell, Molecular & Developmental Biology Program, Sackler School of Graduate Biomedical Sciences, Tufts University, Boston, MA USA; 2 Department of Immunology, Tufts University School of Medicine, Boston, MA USA

**Keywords:** Body patterning, Morphogenesis

## Abstract

Vitamin D3 (vitD3) and its active metabolite, calcitriol (1,25-(OH)_2_D_3_), affect multiple tissue types by interacting with the vitamin D receptor (VDR). Although vitD3 deficiency has been correlated with increased incidence of breast cancer and less favorable outcomes, randomized clinical trials have yet to provide conclusive evidence on the efficacy of vitD3 in preventing or treating breast cancer. Additionally, experimental studies are needed to assess the biological plausibility of these outcomes. The mammary gland of VDR KO mice shows a florid phenotype revealing alterations of developmental processes that are largely regulated by mammotropic hormones. However, most research conducted on vitD3’s effects used 2D cell cultures and supra-physiological doses of vitD3, conditions that spare the microenvironment in which morphogenesis takes place. We investigated the role of vitD3 in mammary epithelial morphogenesis using two 3D culture models. VitD3 interfered with estrogen’s actions on T47D human breast cancer cells in 3D differently at different doses, and recapitulated what is observed *in vivo*. Also, vitD3 can act autonomously and affected the organization of estrogen-insensitive MCF10A cells in 3D collagen matrix by influencing collagen fiber organization. Thus, vitD3 modulates mammary tissue organization independent of its effects on cell proliferation.

## Introduction

Breast cancer remains a major cause of mortality among women worldwide. Epidemiological studies have shown that key stages during breast development are particularly susceptible to the effects of carcinogens. For instance, women aged 10–19 years who were exposed to atomic bomb radiation in Hiroshima during World War II showed an increased incidence of breast cancer at the age of prevalence compared to similarly exposed women aged 35 years and older^1^. Likewise, women exposed to diethylstilbestrol during fetal life have a higher risk of breast cancer compared to unexposed women^2^, and women exposed to DDT in the womb showed a four-fold higher risk of breast cancer in adulthood^3^. Windows of susceptibility have also been observed in rodents; namely, rats exposed to the carcinogen nitrosomethylurea around puberty have a 100% incidence of tumors, but the incidence rate falls to just 10% when exposed to the carcinogen after 90 days of age^4^. Rodents exposed *in utero* to low doses of BPA have also shown a higher incidence of mammary gland tumors in adult life^5,6^. These windows of susceptibility coincide with key milestones of organogenesis and/or tissue remodeling, buttressing the notion that carcinogenesis should be considered as “development gone awry^7^.”

Vitamin D3 (VitD3), and its active metabolite calcitriol (1,25-(OH)_2_D_3_), has been primarily studied in the context of normal and diseased bone development^8^. However, research over the past few decades have shown that vitD3 can affect multiple tissue types, including the mammary gland^8^. For instance, epidemiological data from human populations across different ethnicities and latitudes showed a correlation between vitD3 deficiency and risk of breast cancer incidence, and also between vitD3 deficiency and worse outcomes for breast cancer patients^8–10^. Animal models showed that calcitriol deficiency promotes mammary tumor growth in mice^11^ and vitamin D receptor (VDR) KO animals have a higher mortality rate when they were crossed into a genetically induced breast cancer model background^12^. Moreover, dietary supplementation of vitD3 inhibited tumor growth in xenograft models of mammary and prostate cancers in mice^13^. Although vitD3 supplementation may help with patient outcomes, and post-menopausal women may benefit from a lower breast cancer risk^14^, there is yet no conclusive evidence from randomized clinical trials that validates the efficiency of vitD3 as a therapeutic or preventive option for breast cancer.

Mammary glands of VDR KO mice exhibited a florid developmental phenotype, as exemplified by increased number of terminal end buds, increased ductal extension and lateral branching at puberty^15^, precocious alveologenesis during pregnancy, and delayed post-lactational involution^16^. These developmental processes are largely influenced by the so-called mammotropic hormones, i.e., Estrogen (E), Progesterone (Prg) and Prolactin (Prl). More specifically, E induces ductal elongation, Prg increases lateral branching and Prl stimulates alveologenesis^17^. The VDR KO phenotype in the mammary gland of rodents indicates that vitD3 interacts with these hormones during mammary epithelial morphogenesis. However, the interactions between these mammotropic hormones and vitD3 and the role of vitD3 in mammary epithelial morphogenesis at these developmental stages have yet to be examined.

Studies on vitD3’s action in the mammary gland have largely focused on vitD3’s effects on cell proliferation and apoptosis^8^. Because these studies have mostly utilized 2D cell culture models, they do not represent the 3D environment required for morphogenesis. Additionally, most studies have used a calcitriol dose of 100 nM, a dose chosen based on the deficiency cut-off level for vitD3 in human populations that is determined by measuring the serum levels of calcidiol, the precursor to calcitriol^18^. Therefore, utilizing such a dose assumes a hundred percent conversion rate of calcidiol to calcitriol locally at specific tissues, while not taking into account that calcitriol may elicit a non-monotonic dose response comparable to that observed with other steroid hormones.

In this current study, we have utilized two different 3D cell culture models to investigate vitD3’s role in mammary gland morphogenesis. We observed that calcitriol constrains estradiol’s actions on the organization of mammary epithelial cells, and that this effect is independent of its already well-characterized role in cell proliferation. We also noticed that calcitriol has autonomous effects on mammary ductal organization and describe a novel effect of calcitriol on the organization of collagen fibers. Finally, calcitriol elicited a non-monotonic dose response in mammary epithelial cells, as can be expected from a steroid hormone.

## Results

### Effects of calcitriol on estrogen-induced cell proliferation

**Figure 1:**
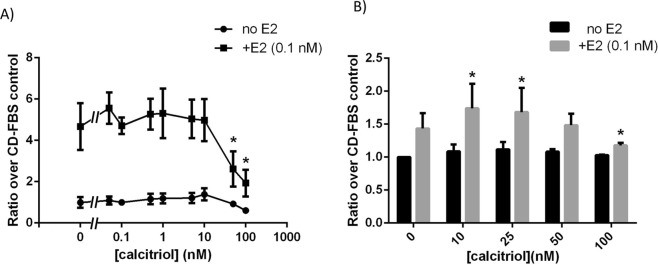
Calcitriol affected total cell yield of T47D cells differently in 2D and 3D culture conditions, and only in the presence of E2 (0.1 nM). (**A**) Calcitriol reduced total cell yield starting at 50 nM and higher doses in 2D culture (data from three independent experiments, n = 4/treatment/experiment; *p < 0.05, one-way ANOVA). (**B**) Calcitriol resulted in increased cell numbers at 10 and 25 nM doses but decreased total cell number at 100 nM dose in 3D culture (n = 6, *p < 0.05 compared to E2, one-way ANOVA).

The human breast epithelial T47D cell line is hormone responsive and proliferates in response to estradiol (E2) in both 2D and 3D cultures^19^. Both in 2D and 3D cultures T47D cells exposed to different doses of calcitriol showed a decrease in cell numbers at the end of a 6-day period only when in the presence of estradiol (E2) (Fig. 1). However, the reduced cell yield was observed at the 50 nM and 100 nM doses of calcitriol alone in 2D (Fig. 1A), whereas a reduced cell yield was observed only at the 100 nM dose in 3D (Fig. 1B). Based on our inverted microscope visualization of dead “floaters” in these cultures, the decrease in cell yield, especially in 2D culture, could be attributed to cell death. It has been previously described that MCF7 cells treated with 100 nM calcitriol, considered supra-physiological^20^, for 48 hours resulted in a decreased percentage of cells in S-phase^21^. To test whether cell cycle arrest contributed to the reduction in cell yield, we utilized a concentration that did not result in cell death (25 nM). Using propidium iodide and flow cytometry, we observed that the percentage of cells in S-phase did not deviate significantly between the 0. 1 nM E2 and 25 nM (+E2) treatments over 4 days. In contrast, there was a marked reduction of cells entering S-phase in the control (CD) group starting at day 2 (Supplementary Fig. 1); this reduction was statistically significant at days 3 and 4. We also observed that the percentage of cells in S-phase in the E2+ 25 nM calcitriol treated group was significantly reduced on day 4 (Supplementary Fig. 1).

In our 3D cultures, we also observed an increase in cell numbers at lower calcitriol doses, such as 10 and 25 nM in 3D culture, an outcome that has not been previously reported (Fig. 1B). Separately, calcitriol activity in these cells was confirmed by performing RT-PCR for *CYP24A1* gene transcripts that showed a dose-dependent increase following calcitriol treatment (Supplementary Fig. 2).

### Effects of calcitriol on estrogen-induced epithelial organization

**Figure 2:**
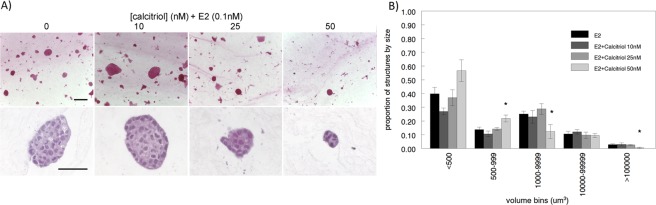
Calcitriol affected the organization of T47D cells in 3D collagen matrix in the presence of 0.1 nM E2, in a dose-dependent manner. (**A**) Calcitriol’s dose-dependent effects observed in carmine-alum stained whole mounted gels (top; scale bar = 200 µm), and in H&E stained FFPE sections (bottom; scale bar = 50 µm). (**B**) 50 nM dose resulted in an increased number of smaller structures with a concomitant decrease in larger structures (n = 6, *p < 0.05, chi-square).

Previous work from our lab had shown that E2 induces T47D cells to form mostly elongated shaped structures when embedded in a 3D rat tail collagen type I matrix^19^. Using this same model, we investigated how vitD3 affects the organization of T47D cells in the presence of 0.1 nM E2; epithelial structures in whole-mounted 3D gels, stained with carmine alum, were imaged using confocal microscopy. As shown in Fig. 2A, calcitriol affected the organization of these cells, mainly by modifying the volume of the epithelial structures in a dose-dependent manner (Supplementary Fig. 3). More specifically, the change in the organization is seen through a re-distribution of different sized structures in the population – the 50 nM calcitriol dose resulted in a higher number of smaller structures whereas the 10 nM dose resulted in a slight, non-statistically significant increased number of larger structures (Fig. 2B). We also observed that there was a concomitant decrease in the number of larger structures in the 50 nM calcitriol (+E2) dose. In contrast, calcitriol alone did not seem to affect these cells when added to medium supplemented with fetal bovine serum (FBS) which was charcoal-dextran (CD) treated to remove estrogens. This outcome suggests that either the interactions with E2 were responsible for the effects observed, or the effect of calcitriol is independent of E2 but was unobservable as the cells do not proliferate in the absence of E2.

**Figure 3:**
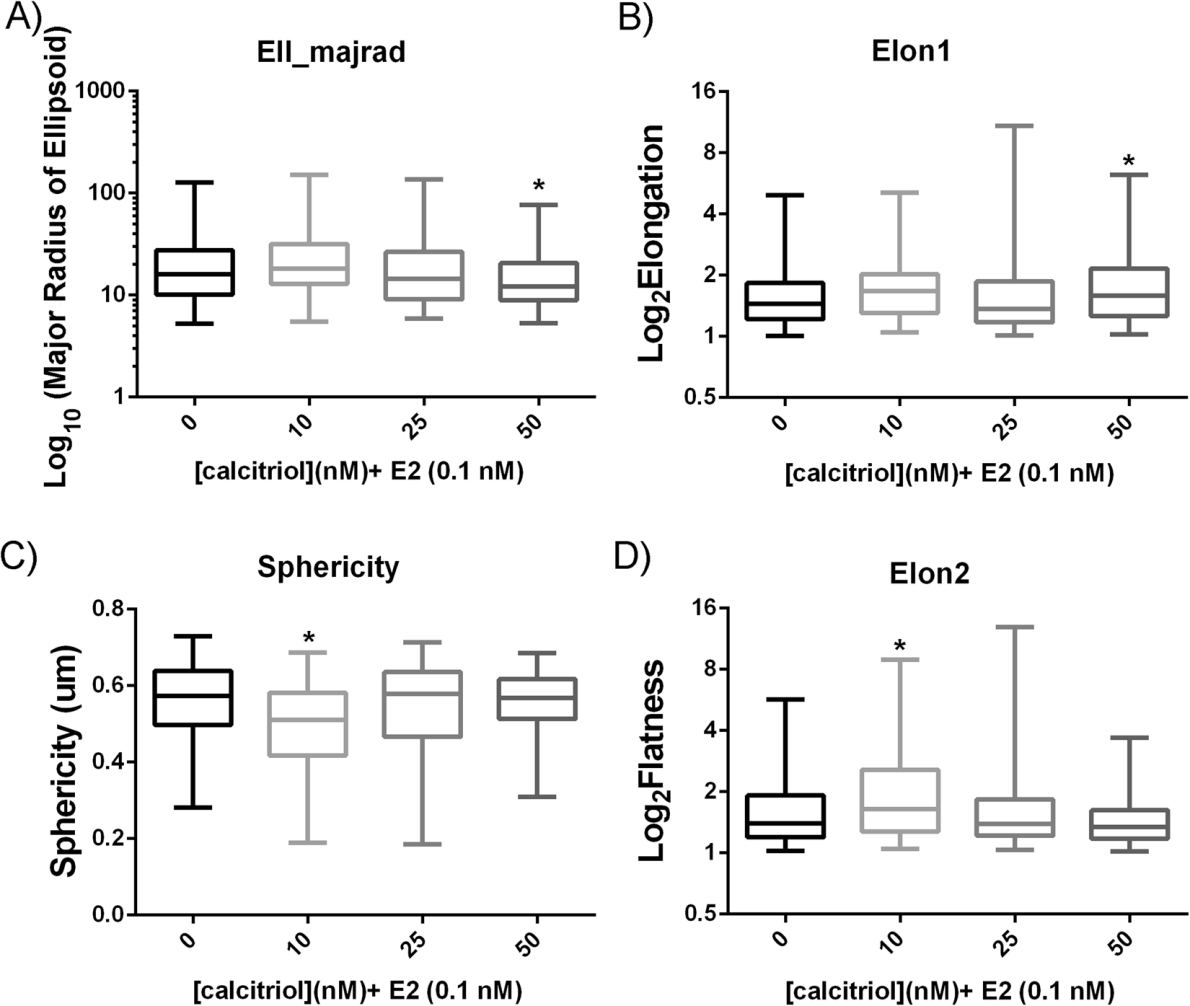
Calcitriol affected physiological parameters of T47D epithelial structures in a dose-dependent manner. Compared to 0.1 nM E2 controls, 50 nM calcitriol resulted in smaller and thinner structures as shown by a decrease in the major radius of the ellipsoid (**A**) and an increase in the elongation ratio (**B**), whereas 10 nM calcitriol resulted in more elongated and wider structures as shown by a decrease in sphericity (**C**) and an increase in flatness ratio (**D**; n = 6, *p < 0.05, Kruskal-Wallis Test).

Further investigation into vitD3’s effects on epithelial organization in 3D cultures using our unsupervised and unbiased analysis software, Software for Automated Morphometric Analysis (SAMA)^22^, revealed that calcitriol affects morphological parameters of the T47D epithelial structures, also in a dose-dependent manner. The 50 nM calcitriol dose resulted in a reduction in the major radius of the ellipsoid (ell_majrad; Fig. 3A) and an increase in the elongation ratio (elon1; Fig. 3B); this observation suggests that this dose results in smaller and thinner structures compared to those induced by 0.1 nM E2. In contrast, the 10 nM calcitriol dose resulted in a decrease in sphericity (Fig. 3C) and an increase in the flatness ratio (elon2; Fig. 3D); in this condition, the T47D structures were more elongated and wider compared to those in the E2 control.

### Calcitriol interferes with estrogen’s transcriptional effects

**Figure 4:**
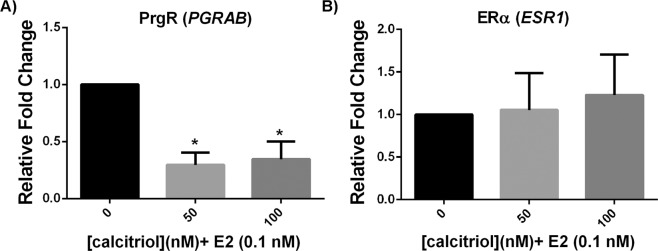
Calcitriol constrains E2’s transcriptional effects without affecting ERα expression. (**A**) Calcitriol decreased E2-induced upregulation of progesterone receptor expression (PrgR, *PGRAB*) at both 50 and 100 nM doses (*p < 0.05 compared to E2, *t*-test). (**B**) Calcitriol does not significantly affect estrogen receptor α (ERα, *ESR1*) expression levels at either 50 or 100 nM dose. Transcripts were measured in T47D cells from 3D collagen gels after 72 hours incubation; RNA was pooled within treatment groups in every experiment (n = 3, Error bars: Standard deviation).

Previous studies have indicated that vitD3 interferes with E2-induced gene expression in cells in culture and human breast tissue^23^. To delineate whether vitD3 was interacting with E2’s activity at the transcriptional level, we extracted RNA from T47D cells in 3D gels and performed RT-PCR to examine the levels of progesterone receptor gene (*PGRAB*) transcripts, which are a reliable indicator of E2’s activity both *in vitro* and *in vivo*. We observed that both 50 and 100 nM calcitriol diminished *PGRAB* induction by E2 by ~60% in T47D cells in 3D culture, as shown in Fig. 4A. This indicates that in our model calcitriol interferes with E2’s activity at the transcriptional level. Published work by others^24,25^ have indicated that calcitriol treatment decreases estrogen receptor α (ERα) expression both at the protein and mRNA levels in MCF7 cells grown in 2D culture. We found that calcitriol treatment (50 or 100 nM, +E2) did not alter ERα mRNA levels in T47D cells in 3D culture (Fig. 4B).

### Estrogen-independent effects of vitD3

**Figure 5:**
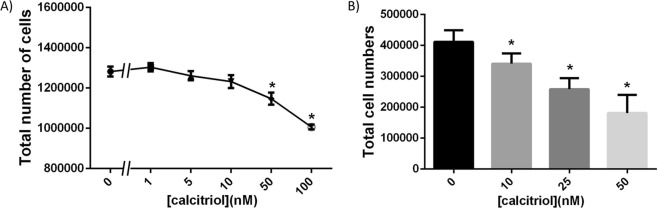
MCF10A cells showed differential sensitivity to calcitriol depending on culture conditions. (**A**) 100 nM calcitriol significantly decreased total cell numbers in 2D culture (data from three independent experiments, n = 4/treatment/experiment), but in 3D culture (**B**), the effects were observed starting at 10 nM (n = 8, *p < 0.05, one-way ANOVA).

MCF10A cells are considered normal breast epithelial cells because they do not form tumors when inoculated into nude/SCID mice^26^ and form normal acinar and ductal structures in 3D cultures^27^. To investigate whether vitD3 has autonomous effects on mammary epithelial cell proliferation and organization, we chose to investigate calcitriol’s effects on MCF10A cells under those conditions. MCF10A cells are ER negative; they respond to calcitriol in a dose-dependent manner as shown by the induction of *CYP24A1* gene transcripts (Supplementary Fig. 4). Because calcitriol exposure resulted in lower cell numbers starting at 10 nM in 3D compared to 50 nM in 2D culture, these cells appear to be more sensitive to calcitriol in 3D culture conditions (Fig. 5). MCF10A cells exhibited a monotonic dose-response relationship in both cases.

### Autonomous effects of calcitriol on epithelial organization

**Figure 6:**
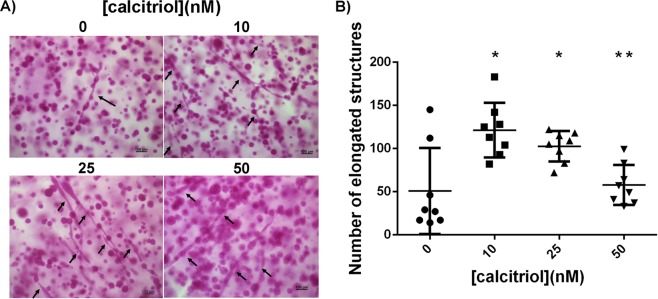
Calcitriol treatment resulted in increased number of elongated structures in a non-linear fashion. (**A**) Carmine-alum stained whole mounts showing elongated structures (arrows; scale bar = 100 µm). (**B**) Quantification of elongated structures in gels show that 10 nM calcitriol resulted in the highest number with consequent decline in higher calcitriol doses (n = 8, *p < 0.05 compared to untreated, **p < 0.05 compared to 10 nM, one-way ANOVA, Tukey’s post-hoc).

Consistent with previous findings^28,29^, MCF10A cells formed mostly acinar structures with few ductal structures when embedded in a rat tail type I collagen matrix and cultured for 2 weeks (Fig. 6A). Interestingly, calcitriol increased the number of elongated structures in 3D culture with the highest number observed at the 10 nM dose, followed by a reduction at higher doses (25 and 50 nM, Fig. 6B).

### Effects of calcitriol on MCF10A epithelial organization

**Figure 7:**
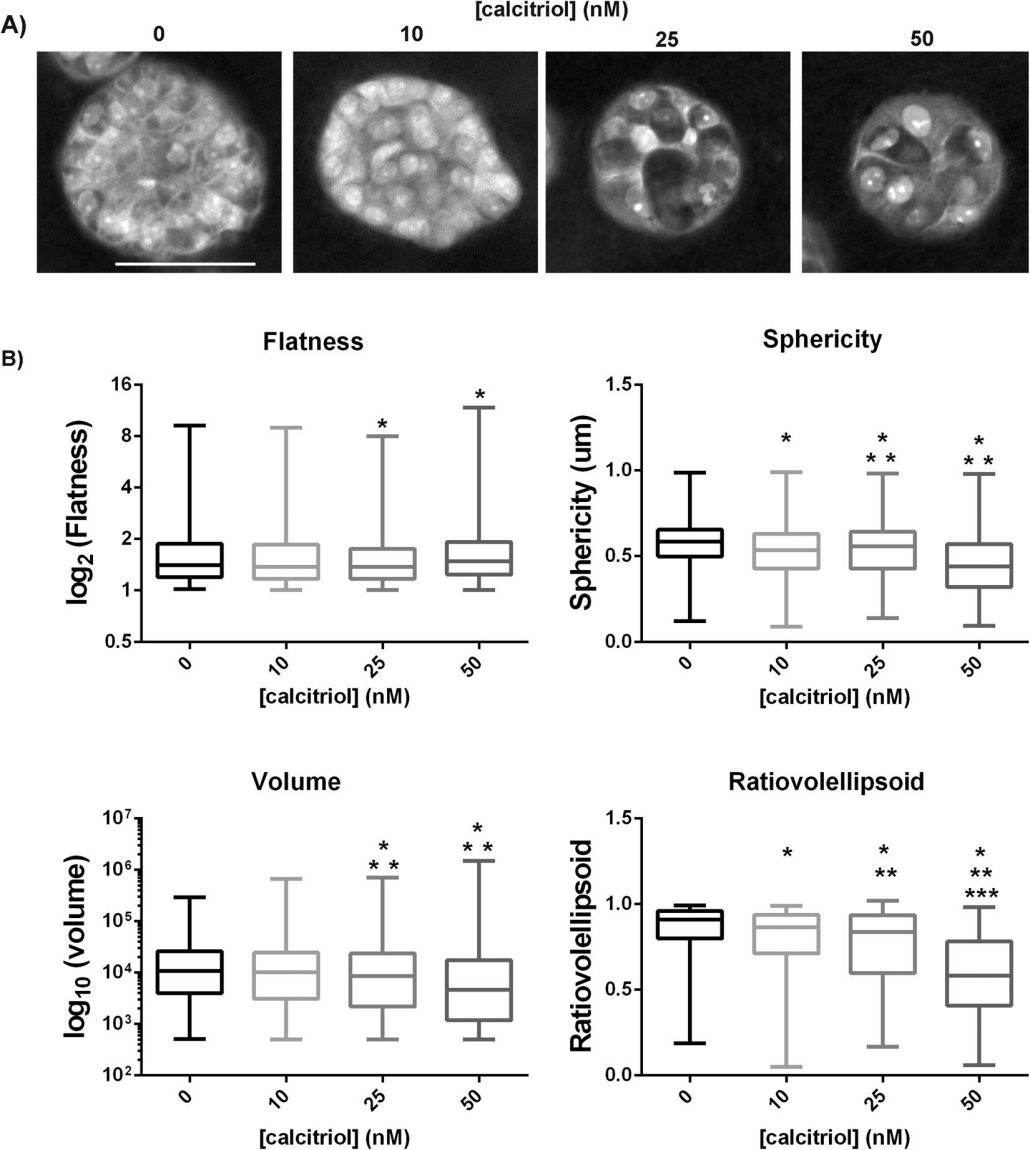
(**A**) Representative 1 µm thick optical sections of MCF10A epithelial structures in collagen type I matrix (scale bar = 50 µm). (**B**) Calcitriol treatment resulted in flatter, less spherical, and less voluminous MCF10A epithelial structures in a type I collagen 3D matrix when cultured for 2 weeks, leading to a change in shape from a sphere to an oblate in a dose-dependent manner (ratiovolellipsoid; *p < 0.05 compared to 0 nM, **p < 0.05 compared to 10 nM, ***p < 0.05 compared to 25 nM Kruskal-Wallis).

Confocal images of MCF10A 3D gels were analyzed for changes in the morphological parameters of the epithelial structures upon calcitriol treatment; representative optical sections (1 µm thick) of MCF10A structures from different treatment groups are shown in Fig. 7A. Exposure to calcitriol resulted in flatter (elon2, Fig. 7B) and less spherical (sphericity, Fig. 7B) structures; these effects were significant at 25 and 50 nM doses for the former and all calcitriol doses for the latter. Calcitriol also resulted in a significant decrease in the volume of these epithelial structures at 25 and 50 nM doses (volume, Fig. 7B), which can be attributed to the increase in flatness with increasing calcitriol doses. The increase in flatness, and the decrease in sphericity and volume of these structures change their shape from a sphere towards an oblate (flattened spheroid). This is reflected in a dose-dependent significant decrease in the ratiovolellipsoid measure (Fig. 7B) - ratiovolellipsoid measures how close to a sphere a structure is and a value of “1” represents a perfect sphere^22^.

### Effects of calcitriol on collagen organization

**Figure 8:**
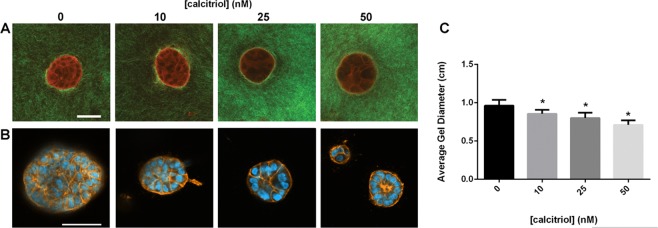
(**A**) Representative SHG images showing calcitriol treatment results in differential collagen organization around epithelial structures in a dose-dependent manner; collagen fibers shown in green (SHG), epithelial structures shown in red (TPEF). (**B**) Visualization of actin filament organization in epithelial structures using rhodamine-labelled phalloidin. (**C**) Calcitriol treatment resulted in an increased contraction of MCF10A gels in a dose-dependent manner (n = 8, *p < 0.05, one-way ANOVA). Scale bars = 50 µm.

MCF10A cells organize collagen fibers in the 3D gels as a prerequisite for organizing into ducts or acini^30^. We investigated collagen fiber organization in 3D gels using picrosirius red staining. Polarized light microscopy of formalin-fixed, paraffin-embedded (FFPE) sections stained with picrosirius red revealed that calcitriol has a non-monotonic effect on collagen fiber organization in this 3D model (Supplementary Fig. 5). Calcitriol at a 10 nM dose reduced the number of organized collagen fibers. In contrast, 25 and especially 50 nM doses showed an increase in the amount of organized collagen fibers. Additionally, at these doses, organized fibers were more uniformly distributed throughout the 3D gels, especially in areas distal from epithelial structures (data not shown). On closer inspection using second harmonic generation (SHG) imaging technique, we observed a higher intensity of SHG signals, which correspond to thicker fiber bundles^31^ around epithelial structures in gels treated with higher (25 and 50 nM) calcitriol doses compared to those in untreated and 10 nM treated gels (Fig. 8A). In gels treated with 10 nM calcitriol, we observed longer, organized collagen fibers around the epithelial structures; the SHG signal intensity was similar to that in untreated gels. Given the flattening phenotype observed in MCF10A structures when treated with calcitriol, and based on the evidence from collagen fiber visualization, we hypothesized that cells must be experiencing greater physical forces at higher calcitriol doses. Cells respond to external mechanical forces by generating endogenous stress through myosin and actin network^32^; we therefore visualized actin filaments using rhodamine-labeled phalloidin. Thicker actin bundles were present in the periphery of the epithelial structures in gels treated with calcitriol (Fig. 8B). These observations may explain why calcitriol treatment results in increased contraction of the 3D gels in a dose-dependent manner after 2 weeks in culture (Fig. 8C).

## Discussion

Despite evidence linking vitD3 deficiency to increased risk of breast cancer and worse clinical outcomes in patients, randomized clinical trials have yet to confirm the efficacy of vitD3 as a preventive or therapeutic option in this disease^8^. Experimentally, while VDR KO mice do not develop tumors spontaneously, mammary glands from these mice exhibit a striking phenotype of excessive and precocious development at key stages^15,16^. This suggests that vitD3 plays an important role in the development of the normal mammary gland. When considering that carcinogenesis results from altered tissue architecture during development (development gone awry) and tissue remodeling^7^, an understanding of the role of vitD3 in this process may provide worthy therapeutic options for breast cancer patients.

The VDR is expressed in the mammary gland at the different stages of postnatal development that are largely influenced by the mammotropic hormones E2, Prg and Prl. These hormones have well-characterized effects on the morphogenesis of the gland; for example, E2 promotes ductal elongation, Prg increases lateral branching and Prl induces alveologenesis^17^. Although the VDR KO phenotype of the gland has been described^15,16^, no reference has been made so far to the interactions between E2, Prg and Prl, and vitD3 in a 3D environment in which morphogenesis takes place. To fulfill this need, we have utilized two different 3D culture models to tease out vitD3’s effects that are either dependent or independent of its interactions with E2. We noticed that calcitriol exhibits a non-monotonic dose response only in 3D cultures, an outcome not previously described. This is in line with current knowledge regarding steroid hormone activity^33^ and also favors the notion that vitD3 functions as a steroid hormone.

In the presence of estrogen in 3D culture, calcitriol increased total cell yield at the 10 nM dose whereas it decreased total cell yield at the 100 nM dose (Fig. 1B). This reduced cell yield can be attributed to cell death given our observation of floater cells in both 2D and 3D cultures. Comparable evidence was found when the role of vitD3 was explored in apoptosis^8,23^; the notion that cell cycle arrest could have been responsible for the reduced cell yield was disproven by our T47D cell cycle analysis experiment (Supplemental Fig. 1). We also observed that calcitriol constrained the effects of E2 on mammary epithelial morphogenesis without affecting total cell yield, more specifically on the organization of epithelial ductal structures in 3D conditions. Consistent with our finding, the mammary glands of *CYP24A1* KO mice, which cannot metabolize calcitriol, exhibit stunted development^34^; Zinser *et al*.^16^ report that VDR KO mammary glands exhibit increased ductal elongation at puberty. Of note, in both of these models, the proliferative capacity of the epithelial cells was not affected. Given that E2 is responsible for ductal elongation during puberty, our results recapitulate vitD3’s activity *in vivo* and confirm that calcitriol at high doses constrains the effects of estrogen on ductal elongation without affecting cell death or proliferation.

The observation that 10 nM calcitriol increased total cell yield in the presence of E2 in 3D culture challenges the commonly reported apoptotic effects of calcitriol^8,23^. To the best of our knowledge, this is the first report of calcitriol increasing breast epithelial cell yield in 3D culture. Previously, only two papers^35,36^ had reported increased cell numbers at calcitriol doses between 0.05 and 0.8 nM in 2D culture. However, we were unable to reproduce these outcomes using cell culture materials from both the same and a different brand, and using two different methodologies – measuring doubling time (data not shown) and total cell yield. We observed a non-significant increase in total cell number in the 0.1 nM calcitriol dose in the presence of estradiol (Fig. 1A).

During puberty, the mouse mammary gland epithelial ducts elongate under the influence of E2. However, only 15–20% of epithelial cells in the gland are ER-positive at that time^37^, with this protein being expressed in the interior luminal layer of epithelial cells and not in the outermost cap layer. A similar pattern of VDR expression in the mammary gland epithelium at puberty has been reported with most expression observed in the trailing edge of the terminal end buds and lesser expression in the cap cells^15^. Therefore, while E2 appears to bind to ER present in 15–20% of epithelial cells during puberty, its effects are observed at the tissue level.

In order to investigate whether vitD3 autonomously affects epithelial cells beyond its interactions with E2, we utilized an estrogen-insensitive 3D culture model. MCF10A cells, considered to portray a “normal-like” behavior *in vitro*, organize into mostly acini and form some ductal structures in the 3D collagen matrix^27^. We observed that vitD3 retains its non-monotonic effects on morphogenesis even in an estrogen-insensitive 3D culture model. We showed that this organization of MCF10A cells is affected in a dose-dependent manner when treated with calcitriol in 3D cultures. These cells showed a greater sensitivity to calcitriol in 3D when compared to 2D cultures, with cell death increasing in a dose-dependent manner upward from a 10 nM dose (Fig. 5).

Mechanical forces are the main mediators of shape during morphogenesis. Previously, we have shown that mammary epithelial cells embedded in a type I collagen matrix manipulate the collagen fibers around them in the process of organizing into complex shapes such as ducts and acini^19,30^; these epithelial cells exert mechanical forces that act on collagen fibers and on neighboring cells. As fibers organize, they constrain the cells on their ability to move and to proliferate^38^. We have also shown that hormones distinctively influence the way epithelial cells organize collagen fibers, and consequently determine the shape of the structures formed^19,31^. Based on these results, we hypothesized that vitD3 would also affect fiber organization, which in turn would affect the mechanical environment of the cells. MCF10A cells treated with increasing concentrations of calcitriol in 3D cultures increased the contraction of gels. Treatment with calcitriol also decreased the number of cells in the gels in a dose-dependent manner. Gel contraction is dependent on the number of cells present in the gel and on the manipulation of collagen fibers by the cells. While the lower number of cells can account for the smaller sizes of structures observed in the 3D gels treated with calcitriol, this association does not explain the increased contraction of these gels. Picrosirius red staining revealed that even though there are fewer cells in the gels at all calcitriol doses compared to untreated gels, there is a more uniform distribution of organized fibers throughout the gel (Supplementary Fig. 5). High resolution imaging using SHG microscopy revealed longer bundles of fibers at all calcitriol doses, and thicker bundles of fibers at 25 and 50 nM doses (Fig. 8A). Organized fibers are responsible for transmission of forces^39^. Cells respond to external mechanical force by generating endogenous stress using myosin and actin networks^32^. Using rhodamine-labeled phalloidin to visualize actin filaments, we observed thicker and pronounced actin networks in the boundaries of the epithelial structures in gels treated with calcitriol (Fig. 8B). This suggests that MCF10A cells experience greater mechanical forces with increasing calcitriol concentration. The increased contraction of the gels (Fig. 8C) can therefore be explained by the transmission of forces generated by the cells on collagen fibers over long distances^40^.

We observed that at the 10 nM dose of calcitriol there was the least amount of fiber bundles, as visualized when using picrosirius red dye and polarized light microscopy (Supplementary Fig. 5) and the highest number of elongated structures (Fig. 6B). On closer observation, the calcitriol treated gels contained a lower number of branched, elongated structures (ductal, tubular) and a higher number of unbranched, elongated structures (cord-like). Additionally, when compared to untreated gels, increase in calcitriol dose resulted in shorter and thinner elongated structures. We have previously reported that MCF10A cells embedded in a collagen type I matrix form ductal/tubular structures in the periphery of the gel^41^. In the 25 and 50 nM calcitriol- treated gels, cord-like structures were observed in the periphery and in the inner areas. In higher resolution images obtained using SHG microscopy, we observed that at the 10 nM dose, while the signal intensity is similar to untreated, the fiber bundles around epithelial structures were longer (Fig. 8A). This may explain the phenotype of epithelial structures observed at the 10 nM dose. We hypothesize that the longer organized bundles facilitate the formation of tubular structures, while at the higher doses of calcitriol, the thicker fibers force the cells to organize in cord-like structures. Additional measurements of local biomechanical parameters in the calcitriol-treated 3D gels are required to test this hypothesis and to elucidate the differential organization of structures depending on the calcitriol dose used.

By definition, all models are simplified versions of the object being modeled. They are used precisely because they reduce the number of variables considered relevant to explain a phenotype. Thus, like all experimental models, 3D culture models have their limitations. For example, the use of established cell lines, which are considered rather stable is dictated by the limitations of using freshly isolated primary cells. Isolated human primary cells are not efficient in forming biologically relevant structures in collagen or ECM matrices *in vitro*; only a small percentage of them express mammotropic hormone receptors and they lose their potential to form structures shortly after being placed in culture^42–44^. Considering these inherent limitations, we have utilized human breast epithelial cell lines such as T47D and MCF10A to create more robust, consistent and complementary models that would still mimic the mammary gland morphogenesis observed *in vivo*.

Future work should incorporate findings from *in vitro* 3D models and test them in an *in vivo* model. To that end, findings that calcitriol constrains the action of estrogen can be incorporated into a mammary gland tissue recombination model between VDR KO and wild type animals to investigate the role of the stromal and epithelial compartments in mediating vitD3’s effects during pubertal development. Similarly, a fetal mammary gland *ex vivo* culture model^45^ can also be utilized to more comprehensively understand the role of vitD3 in early development.

Finally, we have shown that calcitriol, at physiologically relevant doses, have effects beyond cell death and proliferation described in the current literature. This study highlights the role of vitD3 as a morphogen to the extent that calcitriol contributes to the proper shaping of the mammary gland epithelium during development. Disorganization of the tissue architecture during early developmental phases has been shown to contribute to tumor formation in the mammary gland^6,46^. This study provides a more detailed understanding of vitD3’s role in normal mouse mammary gland development and a lead on how vitD3 deficiency might contribute to increased breast cancer risk.

## Materials and Methods

### Reagents

Hydrocortisone, cholera toxin, insulin, Dulbecco’s modified Eagle’s medium (DMEM), Phosphate Buffered Saline (PBS, 10×), propidium iodide, RNase A, and calcitriol were purchased from Sigma-Aldrich (St. Louis, MO). DMEM/F12, L-Glutamine and Trypsin were from Life Technologies (Carlsbad, CA). 17β-estradiol (E2) was purchased from Calbiochem. Fetal bovine and equine sera were supplied by Thermo Fisher (Waltham, MA). Epidermal Growth Factor (EGF) and rat tail type I collagen were from Corning (Tewksbury, MA). E2 and calcitriol were resuspended in ethanol to make 10^−3^ and 10^−4^ M stocks, respectively; calcitriol stocks were stored under nitrogen at −20 °C. RNase A was diluted at 2 mg/mL in distilled, de-ionized water and stored according to manufacturer’s instructions.

### Cell maintenance

Human breast epithelial T47D cells used in this study were cloned from a population originally obtained from Dr. G. Green (U. Chicago). T47D cells were routinely tested to ascertain their estrogen-sensitivity before performing experiments with them^47^. These cells were grown in DMEM containing 5% FBS (propagation medium). When looking at effects of hormones on these cells using 2D culture experiments, we used DMEM/F12 without phenol red with 5% charcoal-dextran stripped FBS (CD-FBS) and 10^6^ U/ml penicillin. When performing 3D culture experiments with the same purpose, we used a mixture of 75% DMEM, 25% Ham’s F12 without phenol red, 7.5% CD-FBS and 2 mM L-glutamine. Human breast epithelial MCF10A cells were purchased from American Type Culture Collection (Manassas, VA) and maintained in DMEM/F12 with phenol red, 5% equine serum, 20 ng/ml EGF, 0.5 µg/ml hydrocortisone, 0.1 µg/ml cholera toxin, and 10 µg/ml insulin. Experiments performed using MCF10A cells used the same medium. In all cases, cells were incubated at 37 °C in 6% CO_2_ and 100% humidity.

### Dose response curves to calcitriol

Dose-response curves to calcitriol in 2D culture were performed in 24-well plates. Cells were seeded at a density of 35,000/well in media and allowed to attach. For MCF10A cells, seeding media was changed to media containing different doses of calcitriol after 24 hours of seeding. For T47D cells, propagation media was removed 48 hours after seeding, and substituted with CD-FBS medium containing different doses of calcitriol with or without E2. After 6 days, cell numbers were determined using the SRB assay^48^.

### Cell Cycle Analysis

T47D cells were seeded in T25 flasks at a concentration of 1 × 10^6^ cells per flask and allowed to attach. After 48 hours, media was replaced with proper treatment media (CD-FBS alone, plus 0.1 nM E2, plus 25 nM calcitriol and 0.1 nM E2) and cells were incubated up to 4 days. Cells were harvested every day through trypsinization and pelleted by centrifuging at 1200 rpm for 3 mins. Cell pellets were washed with 5 mL ice-cold PBS, fixed in 5 mL 70% ethanol with 0.5 mL cold PBS, followed by another PBS wash. Cells were then incubated in the dark for 45 mins with 500 uL RNase A and 500 uL propidium iodide solution (0.1 mg/mL in 0.6% triton-x diluted in PBS). Stained cells were filtered using a 40 µm cell strainer to ensure single cell suspension before analysis on FACSCalibur flow cytometer (BD Biosciences, CA).

### 3D cultures

Collagen type I gels were formulated at a final concentration of 1 mg/mL as described previously^19^. Cells were suspended in the gel solution at a density of 75,000 cells per gel and 1.5 mL of mixture (per well) was poured into 12-well plates. The mixture was allowed to congeal for 30 min at 37 °C, and 1.5 mL of appropriate media was added to each well (CD-FBS media containing E2 +/− calcitriol for T47D, MCF10A media +/− calcitriol). Gels were detached as previously described^41^. Cultures were maintained either for 1 week to measure total cell yield or 2 weeks for morphological assessments. At each endpoint, gels were harvested and processed as described by Speroni *et al*.^19^. Briefly, to estimate cell numbers, cells were extracted by digesting 3D gels with collagenase and then lysed to obtain nuclei that were then counted using a Coulter Z1 particle counter (Beckman Coulter, CA). For morphological assessments, gels were harvested at 2 weeks, fixed with 10% phosphate-buffered formalin, and cut in half. One half was embedded in paraffin, sectioned at 5 um thickness, and stained with Harris’ Hematoxylin and eosin (H&E) for histological analyses. The other was whole mounted and stained with carmine alum to visualize epithelial structures. The diameter of MCF10A gels was measured at the longest axis with a ruler post-fixation and prior to further processing.

### Whole mount analysis

Whole-mounted gels stained with carmine alum were imaged using a Zeiss LSM 800 confocal microscope for automated morphometric analysis as described by Paulose *et al*.^22^. Briefly, ~1 mm^2^ area of the gel periphery was imaged to a depth of ~100 µm. Resulting images were stitched together and analyzed using SAMA^22^ and statistical analyses of morphometric parameters was performed using GraphPad Prism software.

### Picrosirius red staining

FFPE gels were sectioned using a microtome at 5 µm thickness. Gel sections were then rehydrated and stained with picrosirius red solution to visualize collagen fibers and counter-stained with Weigert’s hematoxylin as described by Junqueira *et al*.^49^. Stained sections were observed under polarized light using a Zeiss Axioskop 2 Plus microscope.

### F-actin staining

Collagen gels were harvested and fixed with freshly prepared 2% paraformaldehyde in PBS. The gels were then permeabilized with 0.2% Triton X-100 in PBS followed by washes in PBS and a 1-hour incubation at room temperature with rhodamine-labeled phalloidin (Cytoskeleton Inc., CO) at 1:100 dilution in PBS. The gels were then washed with PBS and counterstained with DAPI (2.5 ug/mL, Sigma-Aldrich), mounted with anti-fade mounting medium (0.02% n-propyl gallate in 80% glycerol) and visualized with Zeiss LSM 800 confocal microscope.

### Second harmonic generation microscopy

SHG images of collagen fibers and two photon excitation fluorescence (TPEF) images of epithelial structures were obtained using a Leica TCS SP8 confocal microscope equipped with a tunable (680–1,300 nm) titanium-sapphire laser (Insight DS+, Spectra Physics, CA). Non-descanned low-noise Leica hybrid (HyD) detectors collected light in the 460 +/− 25 nm (SHG) and 525 +/− 25 nm (TPEF) ranges at an excitation wavelength of 920 nm. In this study, we used a water-immersion 40x objective (NA 1.10, 650 um working distance). Images with a size of 512 × 512 (pixel size 284 × 284 nm) were acquired at an interval of 1 um along the z-axis. While efforts were made to only include one epithelial structure in a 3D stack, it was not always possible especially at the higher calcitriol doses given the contraction of the gels and the consequent crowding of the structures. Reconstruction of 3D images was performed using FIJI (National Institutes of Health, USA).

### Real-Time PCR

Gels were digested using collagenase as described above, and RNA from cells was harvested using Qiagen RNeasy mini kit according to manufacturer’s instructions. Gene transcripts were quantified via RT-PCR using the Luna Universal One-Step RT-qPCR kit (New England Biolabs, MA) in an iQ5 thermocycler (Bio-rad, CA). Transcript levels were normalized to *RPL19* transcripts levels. Primer sequences for genes analyzed are as follows - *PGRAB* 5′-GAGGATAGCTCTGAGTCCGAGGA-3′ (forward), 5′-TTTGCCCTTCAGAAGCGG-3′ (reverse); *RPL19* 5′-TAGTCTGGCTTCAGCTTCCTC-3′ (forward), 5′-TCTGCAACATCCAGCTACCC-3′ (reverse); *CYP24A1* 5′-GAAAGAATTGTATGCTGCTGTCACA-3′ (forward), 5′- GGGATTACGGGATAAATTGTAGAGAA-3′ (reverse); *ESR1* 5′-TAAATGCTGCCATGTTCCAA-3′ (forward), 5′-CCTGTGAGAGAACAGAAACTGG-3′ (reverse).

### Statistics

GraphPad Prism and SPSS software were used for all statistical analyses. One-way ANOVA followed by Tukey’s *post hoc* test were performed to determine differences in the dose-response curves, number of elongated structures in MCF10A gels, MCF10A gel diameters, and percentage of cells in S-phase; one-way ANOVA with *post hoc* Dunnett’s 2-sided *t*-test was performed for cell yields in T47D 3D gels. Kruskal-Wallis test was performed to determine differences in the morphometric parameters of structures. Chi-square analysis was performed to compare distributions of T47D epithelial structures in different volume categories. Unpaired *t*-test with Welch’s correction was used to analyze RT-PCR data. For all statistical tests, results were considered significant at *p* < *0*.*05*.

## Supplementary information

Supplemental Materials

## Data Availability

All data generated or analyzed during the current study are available from the corresponding author on reasonable request.
