# Supplementary material for: Vitamin D3 constrains estrogen’s effects and influences mammary epithelial organization in 3D cultures
Source: Sci Rep. 2019 May 15;9:7423. doi: 10.1038/s41598-019-43308-1 (PMC6520380; doi:10.1038/s41598-019-43308-1)
Supplement: Supplementary file 1 — Supplemental Materials [file 41598_2019_43308_MOESM1_ESM.docx]

**Supplemental Figures**

**Title**: Vitamin D3 constrains estrogen’s effects and influences mammary epithelial organization in 3D cultures

**Authors**: Nafis Hasan^1^, Carlos Sonnenschein^1,2^, Ana M. Soto*^1,2^

^1^Cell, Molecular & Developmental Biology Program, Sackler School of Graduate Biomedical Sciences, Tufts University, ^2^Department of Immunology, Tufts University School of Medicine, Boston, MA.

***Corresponding Author** – Ana M. Soto, ana.soto@tufts.edu

Hasan et al Supplemental Fig 1


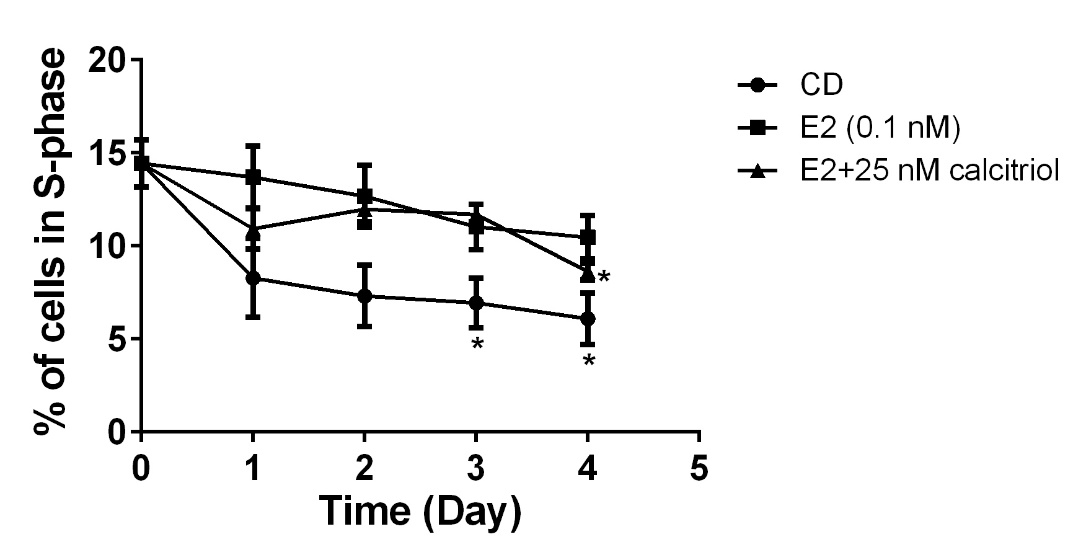


Suppl Fig 1. Cell cycle analysis shows calcitriol does not significantly inhibit cells from entering S-phase. T47D cells treated with CD (CD-FBS), 0.1 nM E2 or 25 nM calcitriol (+0.1 nM E2) were harvested over a period of 4 days and cells were stained with propidium iodide and analyzed for DNA content using flow cytometry. *p<0.05, one-way ANOVA with Tukey’s post-hoc test within treatment groups; error bars: SEM.

Hasan et al. Supplemental Fig 2





**Suppl Fig. 2.** *CYP24A1* induction in T47D cells following calcitriol treatment for 24 hours.

Hasan et al. Supplemental Fig 3





**Suppl. Fig. 3.** Calcitriol at 50 nM concentration lowered the median volume of structures whereas at 10 nM raised the median volume of the same structures (**p*<0.05, Kruskal-Wallis)

Hasan et al. Supplemental Fig 4


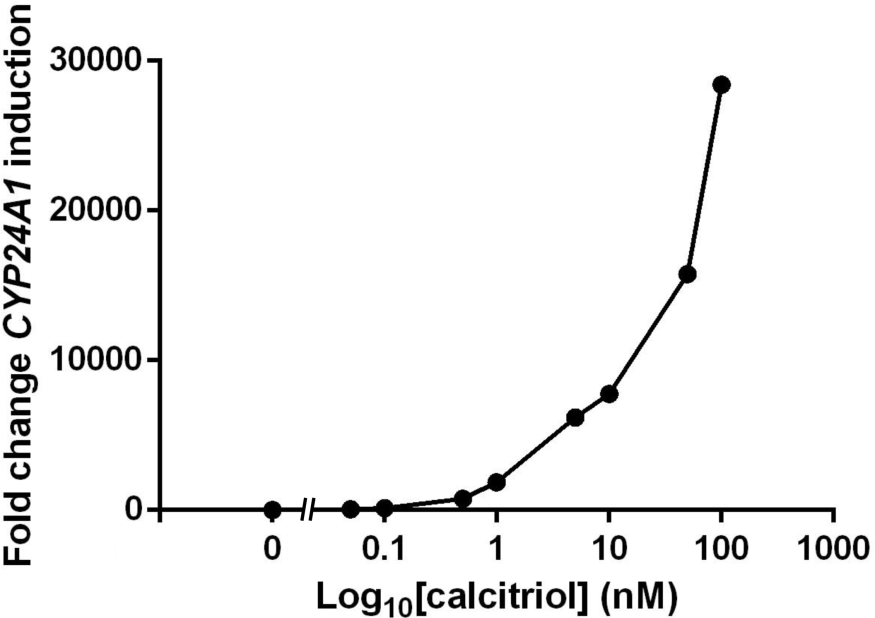


**Suppl Fig. 4**. *CYP24A1* induction in MCF10A cells following calcitriol treatment for 24 hours.

Hasan et al. Supplemental Fig 5


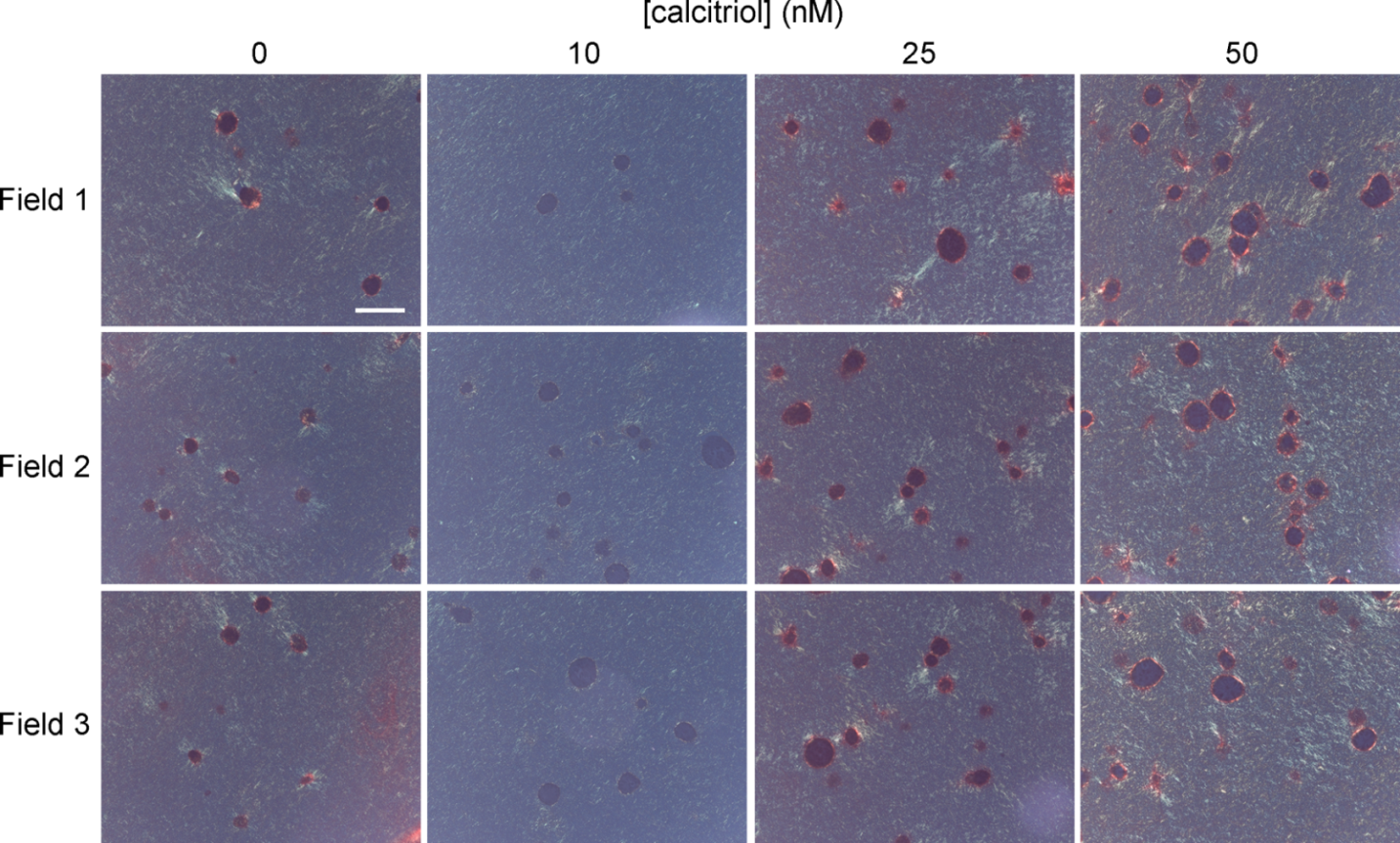


**Suppl Fig. 5.** Calcitriol exposure resulted in a different collagen organization in MCF10A 3D gels. See representative images from three different fields of view for each treatment group (scale bar=100 µm)
